# Laugier-Hunziker-Syndrom: Eine seltene Differenzialdiagnose der mukokutanen Hyperpigmentierung

**DOI:** 10.1007/s00105-021-04845-x

**Published:** 2021-06-25

**Authors:** Sören Korsing, Marcel Boede, Schokufe Ebrahimsade, Katharina Meier

**Affiliations:** 1 Klinik für Dermatologie, Venerologie und Allergologie, Charité – Universitätsmedizin Berlin, Charitéplatz 1, 10117 Berlin, Deutschland; 2 MVZ Hautarztzentrum Tegel, Berlin, Deutschland; 3 Pathologie Dr. med. Ebrahimsade, Berlin, Deutschland

**Keywords:** Orale Hyperpigmentierung, Schleimhautmelanosis, Melanonychia striata, Peutz-Jeghers-Syndrom, Laugier-Hunziker-Baran-Syndrom, Oral hyperpigmentation, Mucosal melanosis, Melanonychia striata, Peutz-Jeghers syndrome, Laugier-Hunziker-Baran syndrome

## Abstract

Das Laugier-Hunziker-Syndrom (LHS) ist durch lentiginöse Hyperpigmentierungen der Mundschleimhaut und Lippen gekennzeichnet. Zusätzlich können longitudinale bzw. striäre Melanonychien und palmoplantare Pigmentläsionen auftreten. Es handelt sich um eine klinische Ausschlussdiagnose. Wir berichten hier über eine 66-jährige Patientin mit LHS. Die klinischen und histopathologischen Merkmale des LHS werden vorgestellt und wichtige Differenzialdiagnosen diskutiert.

## Abstract (en)

Laugier–Hunziker syndrome (LHS) is characterized by lentiginous hyperpigmentation of the oral mucosa and lips. In addition, longitudinal melanonychia and palmoplantar hyperpigmented lesions may occur. LHS is a clinical diagnosis of exclusion. Herein, we report the case of a 66-year-old woman with LHS. The clinical and histopathologic features of LHS are presented and important differential diagnoses are discussed.

## Falldarstellung

### Anamnese

Eine 66-jährige Patientin kaukasischer Abstammung stellte sich mit braunen Pigmentflecken der Mundschleimhaut in unserer Klinik vor. Diese seien erstmals vor ca. 5 Jahren aufgefallen und hätten sich progredient in Anzahl und Größe entwickelt. Weiterhin berichtete die Patientin von braunen Flecken der Finger und Streifen der Fingernägel, die seit ca. 2 Jahren bestünden. Aufgrund einer arteriellen Hypertonie und koronaren Herzerkrankung nahm die Patientin seit 10 Jahren Candesartan und Acetylsalicylsäure ein. Die Einnahme weiterer Arzneimittel wurde verneint. Zurückliegende Strahlen- oder PUVA-Therapien, Traumata, entzündliche Erkrankungen der Haut und Schleimhäute oder Malignome lagen nicht vor. Die Patientin war bis 5 Jahre vor dem Auftreten der Läsionen Raucherin (ca. 10 Packungsjahre). Kindheit und Pubertät seien unauffällig verlaufen. In der Familie bestanden keine Hauterkrankungen. Rezidivierende abdominelle Schmerzen, Diarrhöen, Erbrechen, gastrointestinale Blutungen, Müdigkeit, Blutdruckabfälle oder ein ungewollter Gewichtsverlust wurden verneint.

### Klinischer Befund

**Figure 1:**
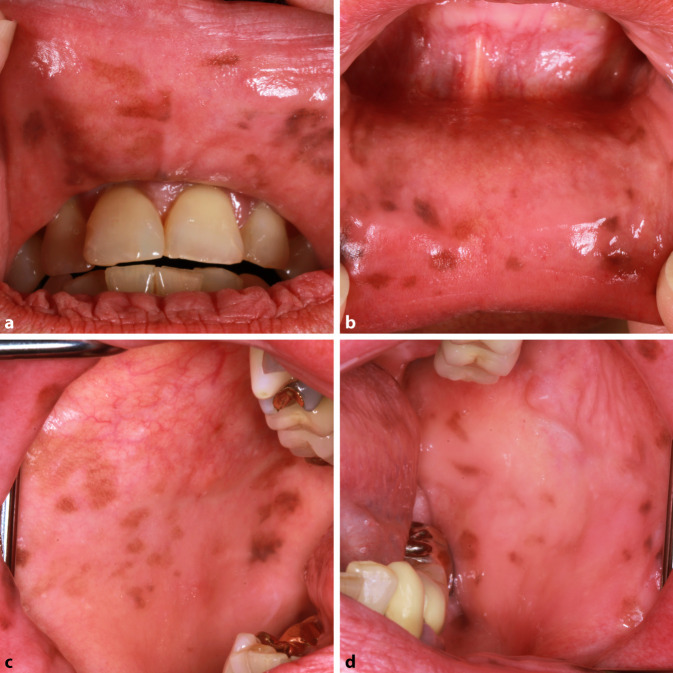

**Figure 2:**
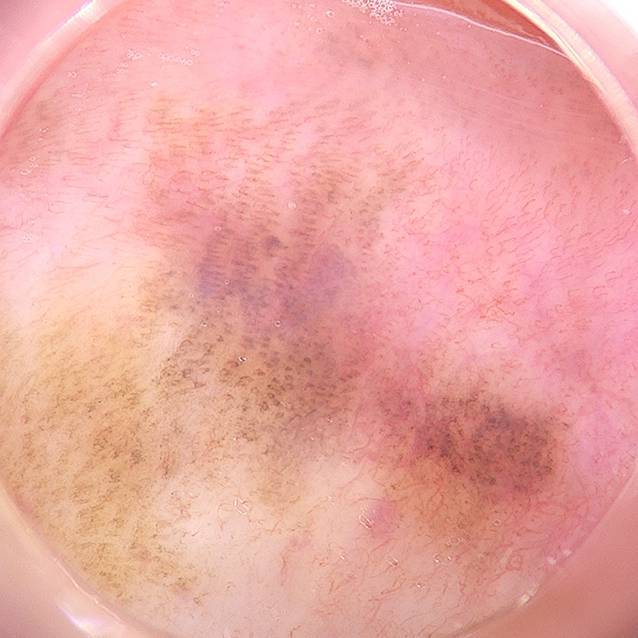

Die Patientin wies einen photobiologischen Hauttyp 2 nach Fitzpatrick auf. Die Inspektion ergab multiple, scharf begrenzte, lentiginöse und polygonal konfigurierte, braune Makulae mit einem maximalen Durchmesser von 10 mm der vorderen Mund- und Wangenschleimhaut. Harter und weicher Gaumen, Gingiva, Zunge und äußeres Lippenrot waren unauffällig. Es zeigten sich 6 goldhaltige Zahnrekonstruktionen. Es lagen keine enoralen Ulzera vor (Abb. 1a–d). In der Dermatoskopie imponierten die Läsionen als regelmäßige, parallel angeordnete, lineare und kurvenförmige, braune Streifen (Abb. 2).

**Figure 3:**
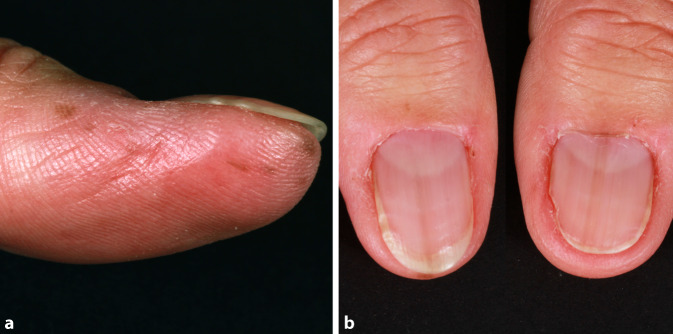

An den Daumen und Zeigefingern fanden sich multiple, scharf begrenzte, lentiginöse, braune Makulae mit einem Durchmesser bis 3 mm (Abb. 3a). Diese stellten sich in der Dermatoskopie als braune, homogene Pigmentierungen der Furchen und Papillarleisten dar. Die Akrosyringien blieben ausgespart. Die Daumennägel wiesen eine Melanonychia striata auf. Das Hutchinson-Zeichen war negativ (Abb. 3b). Der weitere Haut- und Schleimhautstatus sowie der internistische Untersuchungsstatus waren unauffällig.

### Weiterführende Diagnostik

Differenzialblutbild, Leberparameter, Elektrolyte, Kortisol‑, Aldosteron- und Adrenocorticotropinspiegel waren unauffällig. Vor 3 Jahren erfolgte eine Koloskopie ohne pathologischen Befund. Eine Mutation des *STK11*-Gens wurde bereits extern ausgeschlossen.

**Figure 4:**
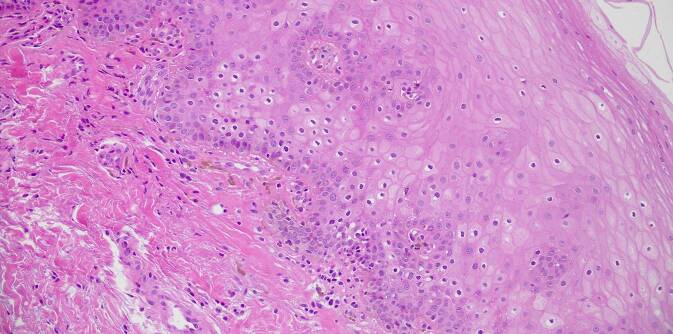

In der histopathologischen Untersuchung einer Läsion der Wangenschleimhaut zeigten sich eine Akanthose, eine basale Hyperpigmentierung sowie subepidermal gelegene Melanophagen. Eine Vermehrung der Melanozyten stellte sich nicht dar (Abb. 4). Eine Biopsie des linken Daumens ergab eine Orthohyperkeratose und Akanthose mit einzelnen, regelrecht differenzierten Melanozyten. Eine erhöhte Anzahl der Melanozyten oder eine Pigmentinkontinenz lag nicht vor.

### Diagnose und Verlauf

Wir stellten die Diagnose eines LHS und klärten die Patientin über dessen Benignität auf. Die Patientin fühlte sich durch die Läsionen nicht kosmetisch beeinträchtigt, sodass keine Intervention erfolgte.

## Diskussion

Das Syndrom wurde erstmals 1970 durch Laugier und Hunziker beschrieben. Das LHS ist durch multiple lentiginöse, braune bis schwarze, orale Makulae gekennzeichnet [6]. Die Schleimhaut der Lippen, Wangen und des harten Gaumens sind Prädilektionsstellen. Selten betroffen sind Zunge, weicher Gaumen und Mundboden. In 50–60 % der Fälle tritt eine Melanonychia striata oder komplette Pigmentierung der Nägel auf. Auf diese Beobachtung machte erstmals Baran aufmerksam, daher auch die Bezeichnung „Laugier-Hunziker-Baran-Syndrom“ [2]. Weitere palmoplantare Hyperpigmentierungen können vorliegen [9]. Die Pigmentläsionen erscheinen spontan und persistieren. Bislang wurden ca. 200 Fälle publiziert. Das LHS tritt gehäuft bei Frauen im 3. bis 5. Lebensjahrzehnt auf [6]. Nur wenige Fälle von Kindern sind bekannt. Eine familiäre Häufung ist selten [7].

Die Ätiologie des LHS ist unbekannt. Pathogenetisch kommt es zur verstärkten Synthese von Melanosomen. Histopathologisch zeigen sich eine Akanthose und Melaninakkumulationen in den basalen Keratinozyten. Melanozyten sind in Verteilung, Anzahl und Morphologie unauffällig. Ein vermehrtes Auftreten von Melanophagen in der papillären Dermis wurde beschrieben [8]. Da es bislang keine Berichte über maligne Entartungen der Pigmentläsionen gibt, gilt das LHS als benigne. Assoziierte Erkrankungen oder Malformationen sind nicht bekannt.

Die Diagnose des LHS wird klinisch gestellt. Andere Ursachen oraler bzw. mukokutaner Hyperpigmentierung müssen ausgeschlossen werden. Bei unserer Patientin sehen wir das Peutz-Jeghers-Syndrom (PJS) als wesentliche Differenzialdiagnose an. Es ist jedoch anzunehmen, dass sich dieses früher manifestiert hätte. Die Hyperpigmentierung des PJS entsteht typischerweise in der Kindheit, die des LHS postpubertär [3, 10]. Zudem können die Pigmentläsionen des PJS perioral, nasal und konjunktival auftreten, was beim LHS bisher nicht beobachtet wurde. Eine Melanonychie tritt beim PJS selten auf. Werden die klinischen Kriterien des PJS erfüllt, liegen zu 90 % Mutationen des *STK11*-Gens vor [1]. Bei unserer Patientin war der *STK11*-Gentest negativ. Kein Verwandter zeigte Anzeichen eines PJS.

Andere Genodermatosen, die mit mukokutanen Hyperpigmentierungen assoziiert sind, kommen als Differenzialdiagnosen des LHS weniger in Betracht, da sich diese frühzeitig manifestieren und weitere klinische Zeichen bieten (Carney-Komplex, Bandler-Syndrom, Noonan-Syndrom mit multiplen Lentigines, McCune-Albright-Syndrom).

Bei den photobiologischen Hauttypen 5–6 nach Fitzpatrick sollte eine ethnisch bedingte Hyperpigmentierung mit in die Differenzialdiagnostik des LHS einbezogen werden.

Mukokutane Hyperpigmentierungen, die durch externe Faktoren verursacht werden, sollten als weitere Differenzialdiagnosen berücksichtigt werden. Die Rauchermelanose tritt bei bis zu 30 % der Raucher auf und betrifft hauptsächlich die mandibuläre Gingiva. Nikotinkarenz führt zum Abblassen der Hyperpigmentierung [5]. Aufgrund der Lokalisation der Schleimhautläsionen, der Finger- und Nagelpigmentierung sowie des Auftretens nach Nikotinkarenz war eine Rauchermelanose bei unserer Patientin ausgeschlossen.

Amalgamhaltige Zahnfüllungen können enorale Makulae verursachen, die durch eine eher blaugraue bis schwarze Farbe gekennzeichnet sind und in direkter Nähe zur Zahnrekonstruktion auftreten (Amalgamtätowierung). Histopathologisch zeigen sich Silbersulfidablagerungen. Amalgam wurde bei unserer Patientin nicht verwendet.

Postinflammatorische Hyperpigmentierungen können als Differenzialdiagnosen des LHS erwogen werden. Mögliche Ursachen stellen Traumata oder entzündliche Haut- und Schleimhauterkrankungen dar. Dies war bei unserer Patientin nicht der Fall. Ebenso gab es keine Hinweise für medikamenteninduzierte Hyperpigmentierungen. Typische Auslöser wären Cotrimoxazol, Antimalariamittel, Tetracycline, Amiodaron, Chemotherapeutika (wie Bleomycin), Antidepressiva, Antikonvulsiva und Schwermetalle [4].

Schließlich kann ein Morbus Addison als weitere Differenzialdiagnose erwogen werden, wobei die Hyperpigmentierungen hier eher flächig imponieren und generalisiert auftreten können. Die fehlende Klinik, der normale ACTH(adrenocorticotropes Hormon)- und Kortisolspiegel und der regelrechte Elektrolythaushalt schlossen einen Morbus Addison bei unserer Patientin aus.

Das LHS ist nicht therapiebedürftig. Patienten sollten über den benignen Verlauf informiert werden, da sie möglicherweise verunsichert sind. Sollten dennoch Zweifel an der Benignität der Pigmentläsionen bestehen, ist eine histopathologische Untersuchung indiziert. Falls eine kosmetische Beeinträchtigung vorliegt, kann eine Laserbehandlung erfolgen.

Das LHS stellt eine wichtige Differenzialdiagnose mukokutaner Hyperpigmentierungen dar. Vermutlich wird das LHS unterdiagnostiziert.

## Fazit für die Praxis

- Das Laugier-Hunziker-Syndrom (LHS) ist durch lentiginöse Hyperpigmentierungen der Mundschleimhaut und Lippen gekennzeichnet.
- Zusätzlich können eine Melanonychia striata und palmoplantare Hyperpigmentierungen vorliegen.
- Differenzialdiagnosen der mukokutanen Hyperpigmentierung (Peutz-Jeghers-Syndrom [PJS], Rauchermelanose, medikamentös induzierte Hyperpigmentierungen u. a.) müssen ausgeschlossen werden.
- Bisher wurde keine Assoziation zum malignen Melanom beschrieben.
